# The tyrosine kinase inhibitor ZD6474 inhibits tumour growth in an intracerebral rat glioma model

**DOI:** 10.1038/sj.bjc.6602108

**Published:** 2004-08-10

**Authors:** M Sandström, M Johansson, U Andersson, A Bergh, A T Bergenheim, R Henriksson

**Affiliations:** 1 Department of Radiation Sciences, Oncology, Umeå University, S-901 85 Umeå, Sweden; 2 Department of Medical Biosciences, Pathology, Umeå University, S-901 85 Umeå, Sweden; 3 Department of Pharmacology and Clinical Neuroscience, Neurosurgery, Umeå University, S-901 85 Umeå, Sweden; 4 AstraZeneca, Sweden

**Keywords:** glioma, ZD6474, angiogenesis, VEGF, tyrosine kinase inhibitor

## Abstract

Malignant glioma is characterised by extensive neovascularisation, principally influenced by vascular endothelial growth factor (VEGF). ZD6474 is a potent inhibitor of VEGF-R2 tyrosine kinase activity, but with additional inhibitory effects on other growth factors. In this study, we have investigated the effects of ZD6474 with regard to tumour growth, neovascularisation, proliferation and apoptosis in the intracerebral rat glioma model, BT4C. ZD6474 (50 and 100 mg kg^−1^) was given as a daily oral gavage. Animals were killed on day 19 and tumour volume was measured. Sections were stained for factor VIII, Ki-67 and for apoptosis. The ability of ZD6474 to inhibit cell growth directly was examined *in vitro*, using the glioma cell line BT4C and the transformed rat brain endothelial cell line RBE4. Cell growth was analysed with fluorometric microculture cytotoxicity assay to quantify the cytotoxic effects. ZD6474 significantly decreased tumour volume compared to controls. Microvascular density increased after treatment with ZD6474, and tumour cell proliferation index was reduced. There was also an increase in tumour cell apoptosis. *In vitro*, the growth of both cell lines was significantly reduced. The results reported justify further experimental investigations concerning the effects of ZD6474 in malignant glioma alone or in combination with other modalities.

Despite extensive treatment efforts, the prognosis for patients suffering from malignant glioma is poor. The search for new treatment modalities as well as improving the efficacy of conventional chemotherapy and radiotherapy is therefore of utmost importance. Malignant glioma is morphologically characterised by extensive pathological neovascularisation, and microvascular density (MVD) is a negative prognostic marker in both low-grade and high-grade glioma (Leon *et al*, 1996; Abdulrauf *et al*, 1998). The neovascularisation is controlled by several different growth regulatory factors of which vascular endothelial growth factor (VEGF) is one of the most important (Yamada *et al*, 1997). Today there are five members in the VEGF family, named A–E. All members signal through three specific receptor tyrosine kinases. VEGFR-1 and -2 are selectively expressed on endothelial cells and VEGFR-3 mostly on lymphatic endothelium, but also on tumour blood vessels (Aprelikova *et al*, 1992; Dvorak, 2002). Vascular endothelial growth factor-A is recognised as the single most important angiogenesis factor with effects on endothelial cell proliferation, protease expression and migration (Machein and Plate, 2000; Ferrara *et al*, 2003). Other growth factors are also of obvious importance, including epidermal growth factor (EGF). Epidermal growth factor and its receptor EGFR contribute to a number of processes important for cancer development and progression, including cell proliferation, apoptosis, angiogenesis and metastatic spread (Ciardiello and Tortora, 2001). Epidermal growth factor receptor is rarely present in normal glial cells, but is expressed in human gliomas. The overexpression of EGFR mRNA, caused by amplification of the erbB-1 gene, has been observed in approximately 40–50% of human glioblastomas (Dunn *et al*, 2000). A truncated and constitutively activated form of EGFR, EGFRvIII, is also frequently seen in glioma and is able to increase VEGF expression in glioma cells (Feldkamp *et al*, 1999). ZD6474, a low molecular weight receptor tyrosine kinase inhibitor (Hennequin *et al*, 2002), inhibits the VEGFR-2 tyrosine kinase with additional effects on VEGFR-3 and EGFR (Wedge *et al*, 2002; Ciardiello *et al*, 2003). In this study, we evaluated the effects of ZD6474 on tumour growth in the intracerebral BT4C glioma model.

## MATERIAL AND METHODS

### Animal model

**Figure 1:**
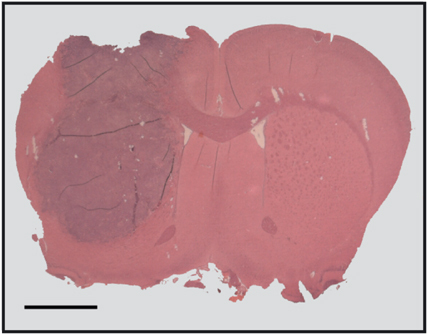
An intracerebral BT4C tumour in the right hemisphere from an animal in the control group, stained with haematoxylin–eosin. Scale bar, 2 mm.

The previously characterised syngenic intracerebral BT4C rat glioma model was used for the *in vivo* experiments in this study (Bergenheim *et al*, 1994; Johansson *et al*, 2000) (Figure 1

). BT4C rat glioma cells growing in log phase were suspended in Dulbecco's modification of Eagle's MEM (DMEM) (Gibco, Paisley, Scotland) supplied with 5% BD IX rat serum to a concentration of 20 000 cells in 5 *μ*l. Inbred BD IX rats were anaesthetised with a 1 : 1 mixture of Hypnorm® (fluanisone 10 mg ml^−1^ and fentanyl citrate 0.315 mg ml^−1^) and Dormicum® (midazolam 5 mg ml^−1^) 0.5 ml (100 g)^−1^ administered as a single i.p. injection. Xylocain® 10 mg ml^−1^ was used for local anaesthesia in the skin of the scalp before incision. With a 22G microsyringe (Unimetrics, Shorewood, IL, USA) fitted to the micromanipulator of a stereotactic frame, 5 *μ*l of the cell suspension was implanted 3.5 mm to the right of bregma at a depth of 4.5 mm (males) and 3.5 mm (females) into the right caudate nucleus under stereotactic conditions. Special care was taken to prevent cellular reflux through the insertion canal and the burr hole in the skull was covered with bone wax. During the implantation procedure, cells were kept on ice and viability was monitored by intermittent tryphan blue staining. After implantation animals were housed in a controlled environment with 12 h light/dark cycles and provided with food and water *ad libitum*. Animals were supervised by an experienced animal keeper who continuously evaluated their overall clinical condition.

Regarding animal welfare, consideration was taken not to expose the animals to unmotivated suffering. The experiments were carried out in strict accordance with the UKCCCR guidelines (Workman *et al*, 1998). The experiment was approved by the local ethics committee for animal research in accordance with the Swedish Animal Welfare Act 1988:534 as last amended by SFS 2002:550, which are adopted in consequence of EC Directive 86/609/EEC (see www.sweden.gov.se/content/1/c6 /02/58/44/51574064.pdf).

For this experiment, 21 animals were used and randomised into three groups with seven animals in each group. The doses chosen is based on published data where it is reported that ZD6474 15–100 mg kg day^−1^ given once daily p.o. to rats for up to 5 weeks was well tolerated, with only small effects on body weight and no adverse effects on clinical condition (Wedge *et al*, 2002). There are no published data on experiments made in intracerebral rat models and in this experiment we chose the doses 50 and 100 mg kg^−1^. These dose levels are within the range recommended by the manufacturers. The control group received 1 ml vehicle, 1% Tween-80 (Merck-Schuchardt, Hohenbrunn, Germany) and the other two groups were treated with ZD6474 50 and 100 mg kg^−1^, respectively. ZD6474 and vehicle were given as a daily oral gavage with disposable animal feeding needles with silicon tip (Scanbur BK AB, Sollentuna, Sweden), starting day 6 after tumour implantation. Animals were weighed twice a week and killed by decapitation on day 19, before they showed neurological symptoms. Blood samples were collected for analysis. The brains were carefully dissected out, placed in phosphate-buffered formalin over night and thereafter fixed in 70% ethanol until paraffin embedded. The total treatment time for the control group and the group receiving ZD6474 50 mg kg^−1^ was 13 days. Owing to poor tolerability in the group that received ZD6474 100 mg kg^−1^, the administration of ZD6474 was ended 1 day earlier. The total treatment time for this group was therefore 12 days. Two animals from the group receiving 100 mg kg^−1^ died before the rest of the animals were killed, one at day 18 and one at day 19.

### ZD6474

ZD6474 was kindly provided from Astra Zeneca, Alderly Park, UK. For details and chemical structure, see Hennequin *et al* (2002). For *in vivo* experiments, ZD6474 was dissolved according to the manufacturer's instructions in 1% Tween-80 (Merck-Schuchardt, Hohenbrunn, Germany) to a concentration of 20 mg ml^−1^. To obtain a uniform suspension, an equal volume of glass beads were added to the mixture and the suspension was then milled over night at room temperature and used within 1 week. For *in vitro* studies, ZD6474 was dissolved in 100% DMSO (Sigma, Stockholm, Sweden) to a concentration of 10 mM. The stock solution was then diluted in cell culture media to achieve different working concentrations.

### Tumour volume

Tumours were sectioned and stained with haematoxylin–eosin. Tumour volume was then measured using a computerised image analysis system. The system used consists of a stereomicroscope (Stemi 2000-C, Carl Zeiss, Jena, Germany) with a high-resolution digital camera (AxioCam, Carl Zeiss, Oberkochen, Germany) under control of KS400 3.0 software (Carl Zeiss, Hallbergmoos, Germany) in a PC computer. Images were analysed using KS400 software. Tumour height and width were measured at largest coronar section. Tumour volume was calculated using the formula for the ellipsoid (*r*1 × *r*2 × *r*3 × *π* × 4/3), where the radius in the sagittal plane was approximated to be the same as the coronal radius (Bergenheim *et al*, 1994).

### Histological analysis of tumours

#### Ki-67 staining and proliferation index

Proliferation index was assessed after immunohistochemical staining for Ki-67 (Scholzen and Gerdes, 2000). Sections chosen for immunohistochemical staining were immersed in citrate buffer (pH 6.0) and irradiated in microwave oven for four cycles of 5 min. Endogenous peroxidase was blocked with 10% H_2_O_2_ in methanol for 15 min, followed by blocking with normal horse serum for 20 min. As primary antibody, a monoclonal anti-rat Ki-67, at 1 : 25 (MIB-5, clone M7248, DAKO A/S, Glostrup, Denmark) was incubated for 1 h at 37°C. After washing in PBS, sections were incubated with biotinylated secondary horse anti-mouse antibody for 30 min. Enzyme conjugate was subsequently added for 30 min. Staining reaction was developed in 3,3′-diaminobenzidine (DAB) (Sigma, Stockholm, Sweden) and mounted using gelatine–glycerol. Cells were manually counted in a standard light microscope (Axiophot, Carl Zeiss, Oberkochen, Germany) and at least 600 nuclei per tumour section were counted. Proliferation index was calculated as the fraction of Ki-67-positive nuclei.

#### Apoptosis

To visualise apoptosis, the Roche *in situ* cell death detection kit, based on the TUNEL technique (Roche Diagnostics Scandinavia AB, Bromma, Sweden), was used. Formalin-fixed, paraffin-embedded tissue sections were treated with proteinase K for 15 min at 37°C and blocked for endogenous peroxidase with 3% H_2_O_2_ in methanol for 10 min. Sections were then incubated for 60 min at 37°C in TUNEL reaction mixture, followed by a 30 min incubation with converter-POD (peroxidase). Finally, the staining reaction was developed in DAB (Sigma, Stockholm, Sweden) and sections were mounted using gelatine–glycerol. Cells were manually counted in standard light microscope (Axiophot, Carl Zeiss, Oberkochen, Germany) avoiding necrotic tumour areas. At least 600 nuclei per tumour section were counted. Apoptosis index was calculated as the fraction of positive stained nuclei.

#### Factor VIII staining and MVD

Vessels in the BT4C brain tumours were immunohistochemically stained for factor VIII and quantified manually using a method originally presented by Weidner (1993). Sections chosen for immunohistochemical staining were permeabilised in 0.1% protease at 37°C for 10 min. After blocking with normal goat sera, sections were incubated for 1 h at room temperature with a primary polyclonal rabbit anti-human factor VIII antibody (DAKO A/S, Glostrup, Denmark) diluted 1 : 400. After washing in PBS, sections were incubated with biotinylated secondary goat anti-rabbit antibody diluted 1 : 200. Endogenous peroxidase was blocked with 10% H_2_O_2_ in methanol for 15 min. Enzyme conjugate was then added for 30 min. The staining reaction was developed in DAB (Sigma, Stockholm, Sweden) and mounted using gelatine–glycerol. Assessment of MVD was performed by manual counting in selected areas with the highest vascular density (hot spots). Each tumour was scanned at low magnification and four hot spots were chosen. All stained objects (blood vessels with and without visible lumina) within a × 200 high-power field were counted using a standard light microscope (Axiophot, Carl Zeiss, Oberkochen, Germany). Each hot spot was counted twice and the arithmetical mean in each section was used to calculate the mean MVD for each tumour, which was used for further statistical analysis. Microvascular density was expressed as number of vessels/ × 200 high-power field (Johansson *et al*, 1999).

### *In vitro* experiments

The nitrosourea induced rat glioma cell line BT4C, kindly provided by professor R Bjerkvig (Bergen, Norway), and the immortalised rat brain endothelial cell line RBE4 (Regina *et al*, 1998), kindly provided by Dr PO Couraud (Neurotech SA, Evry, France), were used for *in vitro* studies. For BT4C assays, cells were grown in cultures as monolayer in DMEM (Gibco, Paisley, Scotland), supplemented with 5% fetal calf serum. Cells were harvested and plated in a volume of 100 *μ*l at 500 cells well^−1^ in microtitre plates. ZD6474 (0–15 *μ*M) was then added to the media. RBE4 cells were grown on calf-skin collagen-coated (Sigma, Stockholm, Sweden) surfaces and maintained in Ham's F10 (Gibco, Paisley, Scotland), supplemented with 5% fetal calf serum. Cells were harvested and plated in a volume of 100 *μ*l at 1000 cells well^−1^ in microtitre plates. ZD6474 (0–10 *μ*M) was then added to the media. Cells were cultured until cell growth was exponential before ZD6474 was added to the media. Plates were incubated at 37°C for 6 days, and the medium was changed after 3 days. To quantify the cytotoxic effects of ZD6474, fluoroscein diacetate (FDA) was used in a fluorometric microculture cytotoxicity assay (FMCA) (Larsson *et al*, 1992). Cells were washed with PBS. PBS (100 *μ*l) containing 10 mg l^−1^ FDA was added to each well and plates were incubated in 37° for 50 min, followed by fluorescence determination using 485 and 538 nm for excitation and emission, respectively.

### Statistics

Values are expressed as median and range. Groups were compared using the Mann–Whitney *U*-test. For curve estimation, quadratic regression was used. For this purpose, the software SPSS 11.0 for Windows was used.

## RESULTS

### Inhibition of tumour growth

**Figure 2:**
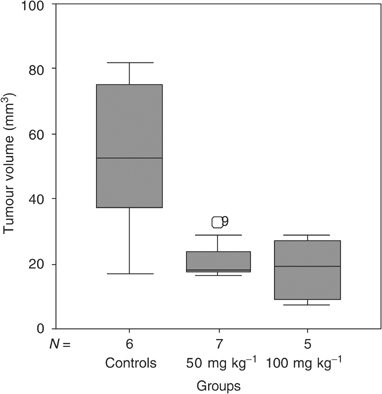
Treatment with ZD6474 50 mg kg^−1^ resulted in significant inhibition of tumour growth from 53 (range 17–82) mm^3^ to 18 (range 16–33) mm^3^ compared with tumours in the control group (*P*=0.032). Treatment with ZD6474 100 mg kg^−1^ resulted in tumour volume 19 (range 7–29) mm^3^ (*P*=0.028 compared to controls). Two cases were excluded due to ‘no tumour take’.

In the BT4C rat model, oral administration of ZD6474 50 mg kg^−1^ once daily for 13 days significantly decreased tumour volume from 53 mm^3^ (range 17–82) to 18 mm^3^ (range 16–33) when compared to controls (*P*<0. 05) (Figure 2

). The higher dose, ZD6474 100 mg kg^−1^ did not provide additional growth inhibitory effect, where tumour volume was 19 mm^3^ (range 7–29) (*P*<0.05 compared to controls), with reservation for the fact that the treatment time here was 1 day less.

### Histopathological analysis of tumours

**Figure 3:**
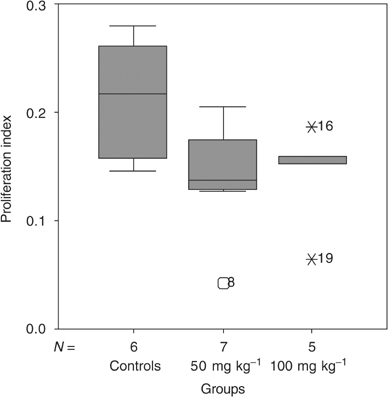
Proliferation index was measured by immunohistochemical staining for Ki-67. Treatment with ZD6474 resulted in reduced proliferation index from 0.22 (range 0.15–0.28) in the control group to 0.14 (range 0.04–0.2) (*P*=0.046) and 0.15 (range 0.06–0.19) (*P*=0.1 compared to controls) in the group treated with ZD6474 50 mg kg^−1^ and 100 mg kg^−1^, respectively.

**Figure 4:**
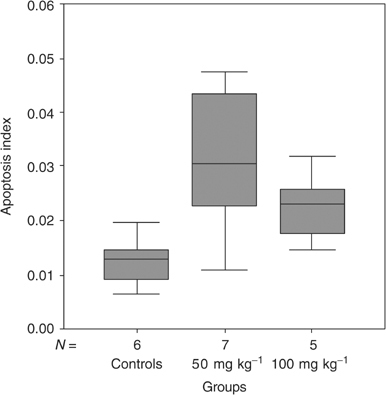
Apoptosis index increased in treated groups. In the control group, apoptosis index was 0.01 (range 0.01–0.02). The group treated with ZD6474 50 mg kg^−1^ had an apoptosis index at 0.03 (range 0.01–0.05) (*P*=0.022), and in the group treated with 100 mg kg^−1^, the apoptosis index was 0.02 (range 0.01–0.03) (*P*=0.022 compared to controls).

**Figure 5:**
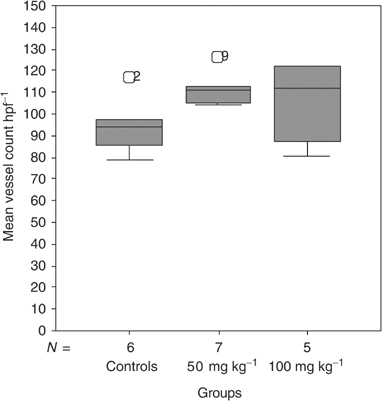
Assessment of mean vessel count was performed after immunohistochemical staining for factor VIII. Treatment with ZD6474 50 mg kg^−1^ resulted in increased mean vessel count from 94 (range 79–117) to 111 (range 104–126) vessels per high-power field (*P*=0.032). The higher dose ZD6474 100 mg kg^−1^ resulted in a mean vessel count of 112 (range 80–122) vessels per high-power field (*P*=0.36 compared to controls).

Treatment with ZD6474 50 mg kg^−1^ reduced the Ki-67 labelling index significantly. The proliferation index in the control group was 0.22 (range 0.15–0.28) compared to 0.14 (range 0.04–0.20) (*P*<0.05) and 0.15 (range 0.06–0.19) (*P*=NS, compared to controls) in the group treated with ZD6474 50 mg kg^−1^ and 100 mg kg^−1^, respectively (Figure 3

). The apoptotic index was increased in the treated groups, with a value in the control group of 0.01 (range 0.01–0.02) compared to 0.03 (range 0.01–0.05) in the group treated with ZD6474 50 mg kg^−1^ (*P*<0.05), and 0.02 (range 0.01–0.03) (*P*<0.05 compared to controls), in the group treated with ZD6474 100 mg kg^−1^ (Figure 4

). The MVD increased from 94 (range 79–117) to 111 (range 104–126) vessels per high-power field (*P*<0.05) in animals treated with ZD6474 50 mg kg^−1^ compared to controls (Figure 5

). In the group treated with ZD6474 100 mg kg^−1^, the MVD was 112 (range 80–122) vessels per high-power field (*P*=NS compared to controls).

### Tolerability

**Figure 6:**
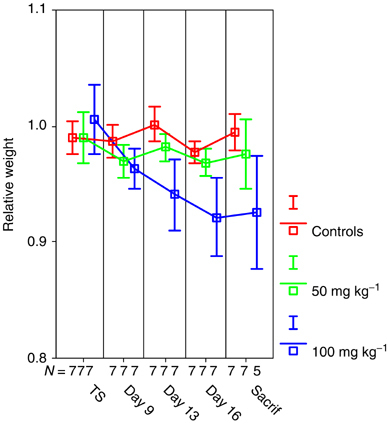
Curves of relative weight among the different groups over time. There was no significant difference in weight between animals in the control group compared to the low-dose group. Animals in the group receiving ZD6474 100 mg kg^−1^ lost significantly in weight. As reported in Material and Methods, two animals in the high-dose group died before the experiment was ended (TS=treatment start, Sacrif=sacrifice).

In BD IX rats, once-daily oral administration of ZD6474 50 mg kg^−1^ for 13 days appeared well tolerated with no visible clinical signs. ZD6474 100 mg kg^−1^ for 12 days made animals lethargic and they lost significant weight (Figure 6

). These animals developed diarrhoea (in some cases blood stained), and autopsy showed marked gastric stocking, signalling delayed gastric emptying. Haematological toxicity was not observed in the experiment. In the group receiving ZD6474 100 mg kg^−1^, the haematocrit and the haemoglobin level were slightly elevated, most likely due to dehydration. In all animals, leucocyte and trombocyte counts were unaffected.

### *In vitro* results

**Figure 7:**
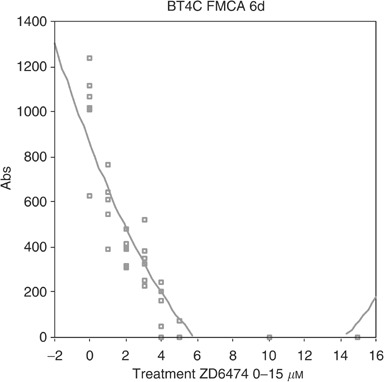
Dose-dependent inhibition of ZD6474 on growth of BT4C tumour cells measured by FMCA with an IC_50_ value of 2.4 *μ*M.

**Figure 8:**
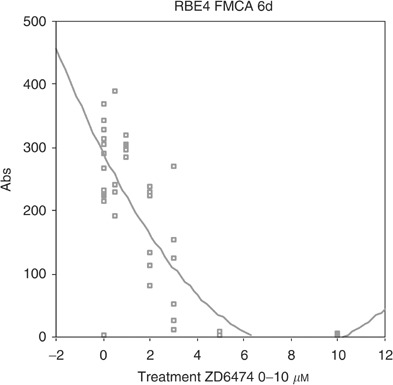
Dose-dependent inhibition of ZD6474 on growth of RBE4 cells measured by FMCA with an IC_50_ value of 2.3 *μ*M.

The ability of ZD6474 to inhibit cell growth directly was examined *in vitro*. ZD6474 significantly and dose dependently inhibited the growth of both BT4C and RBE4. IC_50_ values for inhibition of BT4C was 2.4 *μ*M (Figure 7

), and for RBE4 2.3 *μ*M (Figure 8

).

## DISCUSSION

In this study, we have demonstrated that the VEGFR tyrosine kinase inhibitor ZD6474 significantly inhibits the growth of an intracerebral rat glioma. This finding is novel and, at least to our knowledge, it is the first time growth inhibition of an orthotopic tumour has been reported after ZD6474 treatment. Increased apoptosis and decreased proliferation of tumour cells in treated animals was a finding consistent with the observed growth inhibition in ZD6474-treated animals. *In vitro*, decreased growth of tumour cells as well as of rat brain endothelial cells was observed, which further validates the *in vivo* effects.

Previously, it has been shown that ZD6474 inhibits VEGF signalling and tumour growth in a panel of subcutaneous experimental mouse tumours of different origin (Wedge *et al*, 2002). Growth reductions of 70–100% after ZD6474 50 mg kg^−1^ treatment were reported after 14–28 days of treatment. In our syngenic rat glioma model, growth was significantly inhibited and a tumour volume reduction of 66% was observed after 13 days of treatment with ZD6474 50 mg kg^−1^. The growth reduction observed in our orthotopic glioma model is therefore in accordance with previous reports in other tumour models. However, it must be emphasised that dose levels are not easily translated between different species, and to our knowledge there are no other reports on ZD6474 treatment in rat tumour models. The treatment effects in our intracerebral model also suggest that ZD6474 crosses the blood–tumour barrier and reaches target cells in clinically relevant concentrations. Recently, it has been shown that ZD6474 also has a significant activity in subcutaneous CNS tumour mouse xenografts (Rich *et al*, 2003). Another preliminary report indicates that ZD6474 is inhibiting tumour growth in a mouse model of brain metastasis (Leenders *et al*, 2003). These observations together with the present findings suggest that ZD6474 may be an interesting agent for further investigation in experimental and clinical neuro-oncology.

In the present study, increased apoptosis and decreased proliferation of tumour cells are distinct findings after ZD6474 treatment. The experience from other experimental antiangiogenic treatments is somewhat different. The fragment inhibitors angiostatin and endostatin are both considered to be mainly angiogenesis inhibitors without effects on proliferation of tumour cells (Sim, 1998). It is known that treatment with these angiogenesis inhibitors results in a characteristic pattern with increased tumour cell apoptosis and unaffected tumour cell proliferation (O'Reilly *et al*, 1996; 1997). The observation that ZD6474 also decreases tumour cell proliferation suggests that the inhibition of tumour growth is a result of more than inhibition of angiogenesis alone. This hypothesis is supported by our *in vitro* data where growth of rat brain endothelial cells and tumour cells both were inhibited at similar drug concentrations. Increased apoptosis after exposure to ZD6474 *in vitro* has been reported for different human cell lines (Ciardiello *et al*, 2003). However, in a human lung cancer xenograft (Calu-6), Wedge *et al* (2002) did not observe any change in apoptotic frequency after ZD6474 treatment. In their study, a marked increase in tumour necrosis was reported, suggesting that the antiangiogenic effects are more prominent in this model compared to ours. The literature on the effects of ZD6474 on cell kinetics is so far limited and to our knowledge our study is the first to report an increase in tumour cell apoptosis *in vivo* after ZD6474 treatment.

Proliferation of rat brain endothelial cells was dose-dependently decreased after ZD6474 treatment *in vitro* in our study. This has earlier been reported for human umbilical vein endothelial cells (HUVEC) (Wedge *et al*, 2002) and the effects of ZD6474 on endothelial cells are so far undisputed. However, in our study, RBE4 cells appear to be more resistant to ZD6474 treatment with an IC_50_ more than 30-fold higher than for VEGF-stimulated HUVEC cells, as reported by Wedge *et al*, 2002. The explanation for this difference may be that the endothelial cells in the present study were grown in serum-containing media and not stimulated by VEGF alone. Another explanation may be that brain-derived endothelial cells are more resistant to ZD6474 treatment compared to HUVEC cells derived from human umbilical cord.

The effects of ZD6474 on rat brain endothelial cells *in vitro* could not be translated to a decrease in MVD *in vivo* in the present study. Instead, we observed a slight increase in MVD after treatment with ZD6474 in the BT4C rat glioma model. This result was somewhat unsuspected since it may be assumed that successful antiangiogenic treatment reduces the number of tumour blood vessels. Moreover, vessel density in intradermally transplanted A549 human lung cancer xenografts decreased after ZD6474 treatment (Wedge *et al*, 2002). In a recent report, acute changes in tumour perfusion and permeability were observed *in vivo*, after treatment with ZD6474, using dynamic contrast-enhanced MRI (Checkley *et al*, 2003). Together with the *in vitro* data on rat brain endothelial cells and previous reports, it appears obvious that ZD6474 has the ability to reduce endothelial cell growth *in vivo*. The lack of correlation of treatment effect to a decrease in MVD in the present study may be discussed in several ways. From a biological point of view, vascularisation differs widely between tumour types and models. Microvascular density is also a time-dependent measure and the impact of MVD as a surrogate marker for antiangiogenic therapy response may therefore be discussed. In a review by Hlatky *et al* (2002), MVD as an indicator for antiangiogenic treatment efficacy is questioned . It is stated that MVD may fluctuate during antiangiogenic treatment and it may be assumed that an effective antiangiogenic treatment initially may decrease vessel density and later during the course of treatment a stable state or even an increase in MVD may be predicted as tumour cells drop out. Effects on the vascular component are not independent from effects on the tumour cell compartment and MVD may mainly reflect the ratio of the vascular and tumour cell components. From a methodological point of view, the assessment of MVD is difficult and differences in methodological approaches may also reflect the discrepancy between the present study and the previous report by Wedge *et al* (2002). Therefore, the observed MVD increase in this study does not rule out the possibility that the tumour growth inhibitory effect seen is at least partly due to antiangiogenic effects.

The importance of growth factor stimulation in the progression of malignant glioma is unambiguous. Among angiogenesis factors, VEGF is commonly believed to be the most important positive regulator of angiogenesis in malignant glioma (Machein and Plate, 2000; Mentlein and Held-Feindt, 2003). Hypoxic tumour cells express VEGF and thereby stimulate tumour angiogenesis, one of the hallmarks of high-grade glioma (Plate *et al*, 1992). In the BT4C rat glioma, VEGF is strongly expressed in the invasive tumour border (Lindgren *et al*, 1997). Epidermal growth factor receptor and its main ligands EGF and transforming growth factor alpha (TGF*α*) are commonly overexpressed in malignant glioma (Dunn *et al*, 2000). The rationale to target the VEGF and EGF axis therefore seems obvious in malignant glioma. ZD6474 is designed as an inhibitor of VEGFR-2, but has recently been demonstrated to display additional inhibitory effects on EGFR (Wedge *et al*, 2002; Ciardiello *et al*, 2003). Our *in vitro* data show that the inhibitory effects of ZD6474 on tumour cells did not differ from the effects on endothelial cells *in vitro*, and that proliferation of tumour cells *in vivo* was decreased. This might suggest that the BT4C tumour growth inhibition at least partly involves direct antiproliferative effects, possibly through EGFR inhibition. Further analysis of the expression of growth factors and their receptors from the tumour is required to establish the precise inhibitory mechanisms in this specific tumour model.

The use of tyrosine kinase inhibitors in the clinical setting has so far displayed beneficial effects in a subpopulation of patients with solid tumours. Imatinib (STI571), a receptor tyrosine kinase inhibitor with effects on the bcr-abl tyrosine kinase, platelet-derived growth factor receptor (PDGFR) and c-KIT is now standard treatment in patients with chronic myeloid leukaemia and the rare gastrointestinal stromal tumours (GIST) (Druker *et al*, 2001; Demetri *et al*, 2002). The EGFR tyrosine kinase inhibitor gefitinib (ZD1839) displays activity in several tumours and significant responses and survival benefit in previously treated lung cancer patients are reported (Johnson, 2003). Several other receptor tyrosine kinase inhibitors are under development and in early clinical testing (Bonomi, 2003). However, so far no major contribution has been made from receptor tyrosine kinase inhibitors, besides imatinib for the rare GIST patients, in the treatment of solid tumours. One possible explanation is that the plethora of growth factors active in cancer makes the tumour growth unaffected when only one of the signalling pathways is blocked. The possibility to block transduction of two different pathways stimulating different cell compartments makes ZD6474 interesting for further evaluation in malignant glioma.

To summarise, ZD6474 exerts significant antitumour effects in an intracerebral glioma model with effects on both endothelial and tumour cells. Thus, treatment with ZD6474 could be a novel approach for simultaneous inhibition of both the endothelial cell and the tumour cell components of malignant glioma. The promising experimental results merit ZD6474 for further evaluation in the treatment of malignant glioma.
